# Anticancer Activity of Melittin-Containing Bee Venom Fraction Against Glioblastoma Cells In Vitro

**DOI:** 10.3390/ijms26062376

**Published:** 2025-03-07

**Authors:** Agata Małek, Maciej Strzemski, Lucyna Kapka-Skrzypczak, Jacek Kurzepa

**Affiliations:** 1 Department of Medical Chemistry, Medical University of Lublin, 20-093 Lublin, Poland; jacek.kurzepa@umlub.pl; 2 Department of Analytical Chemistry, Medical University of Lublin, 20-093 Lublin, Poland; maciej.strzemski@umlub.pl; 3 Department of Molecular Biology and Translational Research, Institute of Rural Health, 20-090 Lublin, Poland; kapka.lucyna@imw.lublin.pl; 4 World Institute for Family Health, Calisia University, 62-800 Kalisz, Poland

**Keywords:** bee venom, melittin, MMP-2, MMP-9, TIMP-1, TIMP-2

## Abstract

Previous observations indicating a lower incidence of various types of cancer in beekeepers suggest that greater exposure to stings reduces the risk of cancer development. However, it is not known which of the active compounds of the bee venom (BV) may be responsible for the observed properties. The aim of this study is to evaluate the anti-glioblastoma effect of the main BV fractions. In addition, the effect of BV fractions on the activity of matrix metalloproteinases 2 and 9 (MMP-2 and MMP-9) was assessed. Commercially available BV was divided into three fractions containing one of the main BV components: apamin (fraction #1), phospholipase A_2_ (fraction #2), or melittin (fraction #3). The viability of glioblastoma lines (LN18 and LN229) compared to a physiological line (human MO3.13) was assessed using the MTT. MMP-2 and MMP-9 activity was assessed using gelatin zymography. Tissue inhibitors of metalloproteinases 1 and 2 (TIMP-1 and TIMP-2) levels in cell culture media were measured with the ELISA method. The fraction containing apamin did not show cytotoxic activity up to a concentration of 100 µg/mL. The fraction containing phospholipase A_2_ partially reduced the cells’ viability at a concentration of 100 µg/mL. The greatest activity was demonstrated by the melittin-containing fraction which completely reduced the viability of glioma cells from a concentration of 2.5 μg/mL and inhibited the activity of the assessed metalloproteinases in a dose-dependent manner. After 72 h of incubation, the highest concentrations of TIMP-1 and TIMP-2 (approximately 150 ng/mL and 100 ng/mL, respectively) were observed in the LN229 line. In all tested lines, fraction #3, crude BV, and melittin reduced the secretion of both inhibitors into the medium in a dose-dependent manner. The melittin-containing fraction possessed anti-glioma properties in vitro, suggesting that melittin may be the main anticancer compound of BV.

## 1. Introduction

Glioblastoma multiforme is one of the most dangerous human cancers. Rapid growth, resistance to most chemotherapy drugs, and a spatially limited environment for tumor development make this cancer a major therapeutic challenge [1]. The pharmacological treatment of almost all cancers is based on classic chemotherapy drugs, the role of which is to disrupt the division of tumor cells. In recent years, there has been great interest in biological drugs that may be used in the treatment of various cancers [2]. However, it is also worth remembering that many naturally occurring substances also have proven anti-tumor properties [3]. Among them, bee venom (BV, apitoxin) has been the most thoroughly studied [4,5]. As early as 1979, McDonald et al. reported the results of an analysis of reasons for mortality among qualified beekeepers compared to the rest of the population, revealing that BV has oncoprotective potential, especially for lung cancer [6]. The mechanisms of BV’s antitumor effects, such as its ability to inhibit cancer cell proliferation and growth, influence on the cell cycle, induction of cell death, or effect on extracellular matrix metalloproteinases, still need to be understood at the molecular level [5].

BV contains many biologically active constituents from different groups and classes of chemical compounds, such as peptides, free amino acids, biogenic amines, phospholipids, sugars and their derivatives, aliphatic and aromatic hydrocarbons, and volatile compounds [7,8]. Among the peptides, melittin and apamin are the most abundant constituents of BV. The predominant enzyme is phospholipase A_2_ (PLA_2_) [5]. Melittin, as the main component of BV, represents about 50% of its dry weight, and is followed by PLA_2_, whose content is about 12% [9]. However, the composition of BV is to some extent variable and dependent on various factors, such as the geographic region and season, among others [10]. Melittin is characterized by strong surface activity on cell lipid membranes and hemolytic activity. When melittin is anchored perpendicular to the lipid bilayer, it causes pore formation and membrane disruption, leading to the leakage of intracellular contents [11]. Previous studies have shown that melittin has anticancer activity. By inducing the upregulation of reactive oxygen species (ROS) production, increasing intracellular iron concentration, damaging mitochondria, affecting intracellular pathways, and disintegrating the cell membrane, melittin leads to the apoptosis of cancer cells (this topic is discussed in detail in [8]). Apamin is a basic peptide with neurotoxic effects in the mammalian spinal cord, leading to muscle spasms. Furthermore, apamin has a selective inhibitory effect on calcium-dependent potassium channels [5,12]. PLA_2_ from BV is a hydrolytic enzyme that has the ability to specifically cleave the ester bonds of phospholipids at the sn-2 position, the products of which are lysophospholipids [13]. Moreover, unsaturated fatty acids, as products of the hydrolysis of the enzyme, are also precursors to the synthesis of inflammatory mediators such as prostaglandins and leukotrienes; therefore, PLA_2_ is the most important apitoxin allergen. Melittin may increase PLA_2_ activity, indicating a synergistic interaction between these two venom components regarding lytic activity on cell membranes [5,8,14].

When searching the PubMed database for publications related to the words “glioblastoma” and “bee venom”, only nine items appear. A recent study by Chahla et al. in 2024 showed the cytotoxic effect of *A. mellifera syriaca* BV on human glioma U87 cells. The authors point out that the destructive effect of BV on the cell membrane occurs to a greater extent than the induction of apoptosis in vitro. However, an in vivo experiment conducted on mice showed that the activation of the apoptosis pathway under the influence of BV was expressed by an increase in the expression of caspase 3, the main executor of this process [15]. In another study, melittin and cisplatin showed synergistic cytotoxic activity against human glioma DBTRG-05MG cells. This effect was manifested by the induction of apoptosis via the stimulation of the transient receptor potential melastatin 2 (TRPM2) cation channel [16].

Matrix metalloproteinases (MMPs) are a group of proteolytic enzymes responsible for degrading extracellular matrix (ECM) proteins and vascular basement membrane. They are responsible for numerous processes, both physiological and pathological, in humans. MMPs play also a significant role in the development of various cancers and connective tissue diseases [17]. Dysregulation of MMPs is associated with angiogenesis and the invasion and metastasis of tumors [18]. Two enzymes among the MMPs, MMP-9 and MMP-2 (gelatinases), play a key role in several types of cancer. MMP-9 is involved in the endothelial–mesenchymal transition (EMT), a process that determines cells’ ability to migrate. Furthermore, MMP-9 also modulates tumor-associated inflammation through cytokines and their receptors [18]. Decreased MMP-2 expression has been related to reduced proliferation, clonal growth, and metastasis of cancer cells. Reduction in the levels of these metalloproteinases also promotes apoptosis of tumor cells [19]. Both gelatinases are most strongly inhibited by their natural inhibitors, small peptides, tissue inhibitors of metalloproteinases (TIMP-1 and TIMP-2) [20]. Disruption in the balance between MMPs and TIMPs has been associated with the pathogenesis and progression of numerous diseases, including cancer [21]. Our previous studies were conducted using artisanally obtained BV. The experiments showed that BV exerted a cytotoxic effect on 8-MG-BA and GAMG human primary glioblastoma cell lines. The effect of a reduced secretion of MMP-2 and MMP-9 into the medium under the influence of incubation with BV was also demonstrated [22]. A previous study on other types of cancer has shown that melittin suppressed tumor necrosis factor (TNF)-induced MMP-9 activity by inhibiting the phosphorylation of p38 and ERK1/2 in human aortic smooth muscle cells [23]. Another study has shown that high doses of melittin attenuated the mRNA and protein expression of MMP-2 and MMP-9 in osteosarcoma 143 B cells, leading to reduced tumor metastasis [24].

However, to our best knowledge, previous studies have focused on assessing the activity of crude BV or the activity of isolated components. They have not focused on the activity of individual BV fractions containing only some of their biologically active components (see ref. [8] for a deeper analysis of the issue). Before BV can find clinical use as a compound potentially used in the treatment of glioblastoma, in vitro studies must first determine which components of the venom may be responsible for the postulated anticancer properties. Therefore, our objectives were as follows: (1) evaluation of the cytotoxic properties of BV against selected glioblastoma multiforme lines, (2) chromatographic separation of BV into three fractions which will each contain one of the main natural components of BV: apamin, phospholipase A_2_, and melittin, (3) assessment of the cytotoxic properties of the obtained fractions, (4) evaluation of the obtained fractions’ effect on the activity of MMP-2 and MMP-9 secreted into the culture medium.

## 2. Results

### 2.1. Chemical Composition of Isolated Fractions

**Figure 1:**
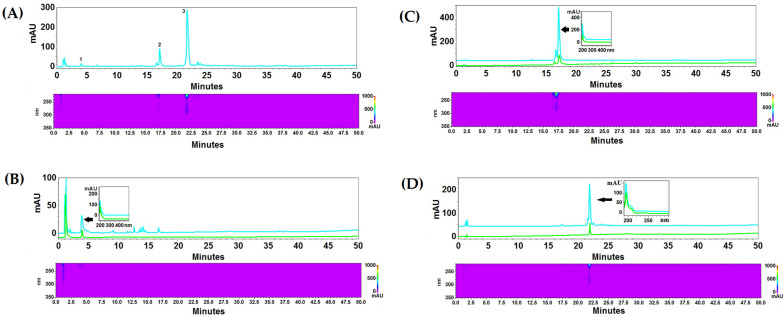
HPLC-PDA chromatograms of crude BV (**A**): 1—apamin, 2—phospholipase A_2_, 3—melittin. The BV fractions: (**B**) fraction #1 with apamin, (**C**) fraction #2 with phospholipase A_2_, (**D**) fraction #3 with melittin. The analysis was performed on an RP18 reversed-phase column Kinetex (10 cm × 4.6 mm i.d., 2.6 μm particle size), at 20 °C, with a flow rate of 1 mL/min. A mixture of acetonitrile with 0.025% of TFA (solvent A) and water with 0.025% of TFA (solvent B) were used as a mobile phase. The compounds were separated using gradient elution with the following program: 0–5 min 10% A, 90% B; 5–25 min 10–50% A, 90–50% B; 25–40 min 50% A, 50% B; 40–65 min 50–100% A, 50–0% B. Blue lines represent chromatograms of crude BV and fractions, green lines represent the individual reference substances.

Fractionating 1 g of BV yielded 154 mg of fraction A, 66 mg of fraction B, and 48 mg of fraction C. Chromatographic analysis revealed the presence of a peak with a retention time of 4 min and a UV–vis spectrum corresponding to apamin standard in fraction A, a time of 17 min and a spectrum corresponding to phospholipase A_2_ in fraction B, and a retention time of 22 min and a spectrum corresponding to melittin standard in fraction C. The percentage of apamin in fraction A was 8.44% which indicates the presence of a significant amount of other compounds. Fraction B contained almost pure phospholipase A_2_ (approximately 96%), while in fraction C, melittin was the dominant compound (over 69%) (Table 1). HPLC-PDA chromatograms of the venom and the fractions obtained are shown in Figure 1.

### 2.2. Cytotoxic Effect of BV Fractions vs. Crude BV and Melittin Standard

**Figure 2:**
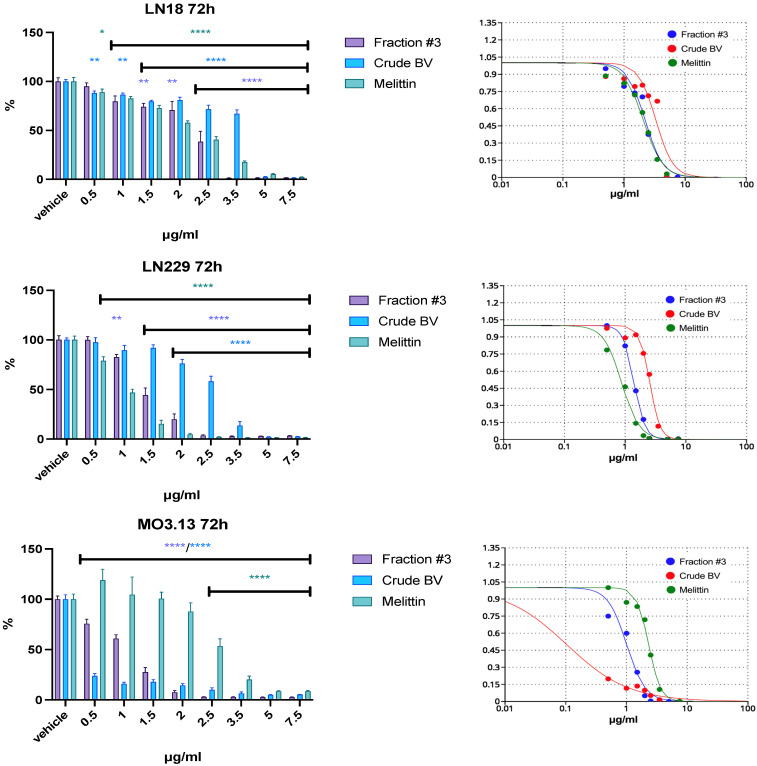
The effect of fraction #3, crude BV, and melittin standard on LN18, LN229, and MO3.13 cell lines’ viability after **72 h** of incubation. Vehicles were set as 100% ± SEM. ANOVA multiple comparison with post hoc Dunnett’s test. * *p* < 0.05, ** *p* < 0.01, **** *p* < 0.0001 compared to controls (vehicle).

The fraction containing apamin (fraction #1) did not present or presented weak cytotoxic activity up to a concentration of 100 µg/mL against all applied cell lines, glioblastoma and control. Fraction #1 had the most pronounced cytotoxic effect against control cells (MO3.13) after 72 h of incubation. Fraction #2 (containing PLA_2_) showed the strongest, clearly incubation-time-dependent cytotoxic effect, also on MO3.13 cells. After 72 h of incubation at a concentration of 10 µg/mL, this fraction completely reduced the viability of MO3.13 cells. At the same duration of incubation, the viability of LN18 and LN229 cell lines was reduced to approximately 25% of the initial value only at a concentration of 100 μg/mL. The greatest decrease in cell viability among the obtained BV fractions was revealed by the melittin-containing fraction (fraction #3). Therefore, lower concentrations in the range of 0.5–7.5 μg/mL were used for this fraction in the next stage of the study. Because fraction #1 and fraction #2 did not show a strong cytotoxic effect on glioblastoma cells at any of the applied incubation times, both fractions were excluded from further analysis. Fraction #3 reduced the viability of both glioblastoma cell lines at a concentration ranging from 2.5 to 3.5 μg/mL during each of the tested incubation periods. After a 24 h incubation, fraction #3 reduced the viability of LN18 and LN229 cells at concentrations approximately two times lower than the concentrations reducing the viability of physiological human MO3.13 cells. However, when the incubation period was extended to 48 h or 72 h, a reduction in MO3.13 cells’ viability was observed at lower fraction #3 concentrations compared to glioblastoma cell lines. The summary results of IC_50_ are provided in Table S1. It is worth noting that the IC_50_ of crude BV for the MO3.13 line was an order of magnitude lower compared to the other IC_50_ values. The effect of BV fraction #3, crude BV, and melittin after a 72 h incubation period is shown in Figure 2. The results of the cytotoxic effect all BV fractions vs. crude BV and melittin standard after 24 and 48 h of incubation as well as fractions #1 and #2 at 72 h are provided in Figures S1–S5 in Supplementary Materials.

### 2.3. Effect of Fraction #3, Crude BV, and Melittin on Gelatinolytic Activity

**Figure 3:**
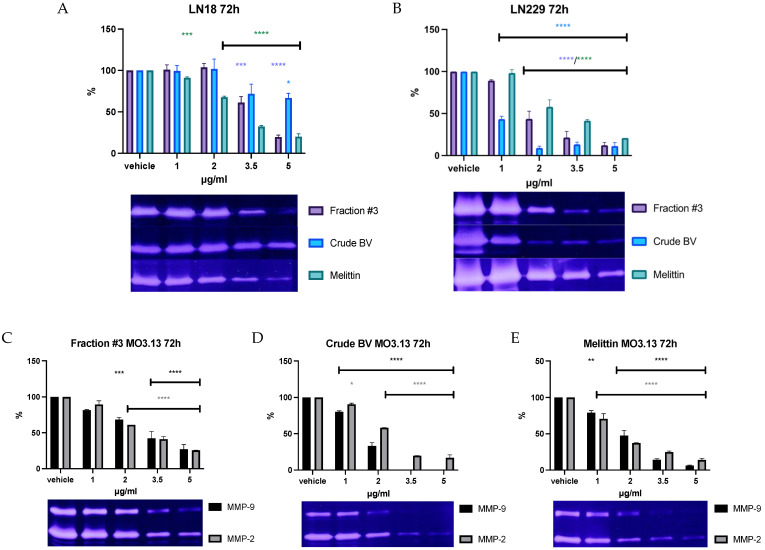
The effect of fraction #3, crude BV, and melittin standard on **MMP-2** secretion from LN18 (**A**) and LN229 (**B**) cell lines, as well as the effect of fraction #3 (**C**), crude BV (**D**), and melittin (**E**) on **MMP-9** and **MMP-2** secretion from MO3.13 cell line after **72 h** of incubation. Enzyme activities were expressed as % of measured optical density and it was set as 100% for the vehicle. Figures are shown together with representative zymograms. ANOVA multiple comparison post hoc Dunnett’s test. * *p* < 0.05, ** *p*< 0.01, *** *p* < 0.001, **** *p* < 0.0001 compared to controls (vehicle).

The gelatinolytic activity coming from MMP-2 was detected in media collected from all analyzed cell lines. The clear activity of MMP-9 was detected only in medium from MO3.13 cells. The dose-dependent effect of fraction #3, crude BV, and melittin on the reduction in MMP-2 secretion was observed in all analyzed cell lines. Moreover, MMP-9 activity was also decreased in MO3.13 after incubation with fraction #3, crude BV, and melittin through all applied periods. The results of zymographic analysis after 72 h of incubation together with representative zymograms are shown in Figure 3. The results of zymographic analysis after 24 and 48 h of incubation are provided in Figures S6–S9 in Supplementary Materials.

### 2.4. Effect of Fraction #3, Crude BV, and Melittin on TIMP-1 and TIMP-2 Secretion

**Figure 4:**
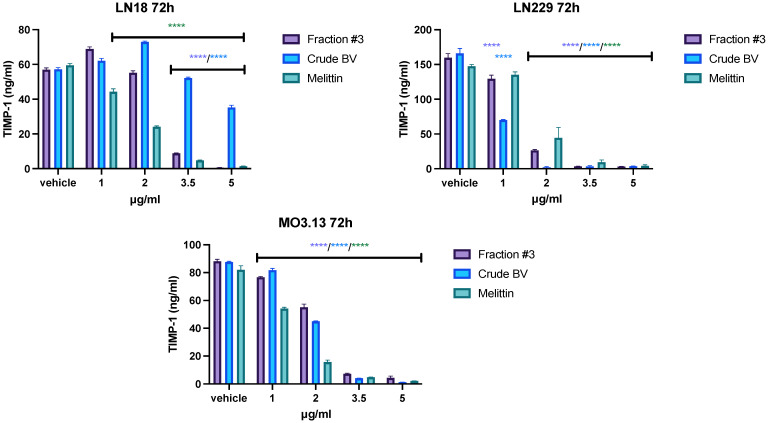
The effect of fraction #3, crude BV, and melittin on **TIMP-1** secretion from LN18, LN229, and MO3.13 cell lines after **72 h** of incubation. Mean TIMP-1 level ± SEM. ANOVA with multiple comparison post hoc Dunnett’s test. **** *p* < 0.0001 compared to controls (vehicle).

**Figure 5:**
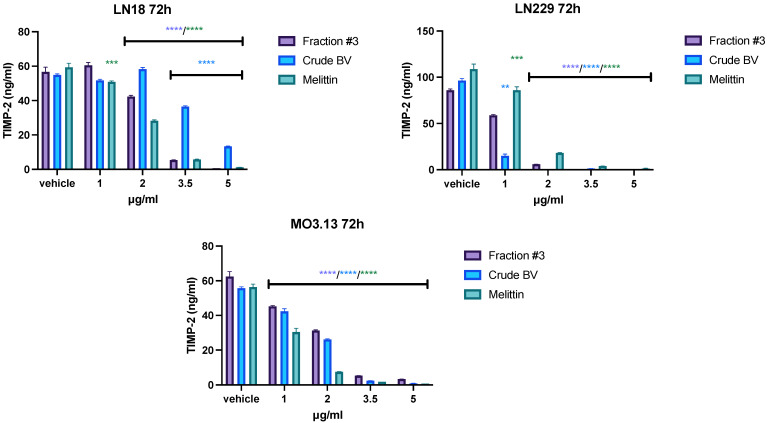
The effect of fraction #3, crude BV, and melittin on **TIMP-2** secretion from LN18, LN229, and MO3.13 cell lines after **72 h** of incubation. Mean TIMP-2 level ± SEM. ANOVA with multiple comparison post hoc Dunnett’s test. ** *p* < 0.01, *** *p* < 0.001, **** *p* < 0.0001 compared to controls (vehicle).

Fraction #3, crude BV, and melittin all reduced the secretion of TIMP-1 and TIMP-2 into the medium in all tested lines. However, the observed effect varied depending on the cell type. First, the highest TIMP-1 concentration, reaching over 150 ng/mL after 72 h of incubation, was obtained in the LN229 line. In physiological cells of the MO3.13 line, the average concentration of TIMP-1 in the post-culture medium did not exceed 100 ng/mL, and in the case of LN18 cells it did not exceed 60 ng/mL. Similarly, the highest TIMP-2 concentration was observed in the LN229 line (average 100 ng/mL). For the LN18 and MO3.13 lines, the average TIMP-2 concentrations after 72 h of incubation were around 660 ng/mL. Secondly, the most pronounced effect of inhibiting the secretion of TIMP-1 and TIMP-2 was observed in the case of the LN229 line, in which crude BV completely inhibited the secretion of both tissue inhibitors at a concentration of 2 μg/mL. For comparison, the concentration of 2 μg/mL of crude BV did not reduce TIMP-2 secretion in LN18 cells, while TIMP-1 secretion slightly increased after 72 h of incubation. In all tested cell lines, the concentration of 3.5 μg/mL of fraction #3, crude BV, and melittin completely inhibited the secretion of TIMP-1 and TIMP-2. The results of the concentrations of both tissue inhibitors after an incubation time of 72 h are presented in Figure 4 and Figure 5. The remaining incubation times (24 h and 48 h) are presented in Figures S10–S13 in Supplementary Materials.

## 3. Discussion

BV has been used as a treatment for many diseases for hundreds of years. To this day, apitherapy is a branch of medicine used in some countries [25]. The use of BV for the reduction of tumor development is one of the main topics of venom research. The reports from basic studies as well as some clinical observations suggest the effectiveness of BV in the treatment of cancer [8]. Like many substances of natural origin, BV is a mixture of various compounds [5]. Therefore, determining which of the constituents is directly responsible for the observed effects is not easy. All the more so because when acting in the environment of other compounds, different types of dependencies may occur between them, changing the activity of each compound.

The main active components of BV are melittin, apamin, and phospholipase A_2_ [26,27,28]. In our study, we separated BV into three main fractions, selecting the parameters so that each fraction contained one of the above-mentioned active compounds which was confirmed chromatographically. The effect of the separation was to obtain fractions containing one of the main components along with other, less numerous compounds that were transferred to a specific fraction. The percentage content of melittin, apamin, and phospholipase A_2_ in individual fractions was confirmed in the chromatographic separation of each fraction.

Analysis of cell viability showed that BV fractions containing apamin (#1) and PLA_2_ (#2) slightly reduced the viability of both glioblastoma cell lines even in concentrations up to 100 μg/mL. Fraction #1 reduced MO3.13 cell viability to just below 50% only after 72 h of incubation. Fraction #2 (containing PLA_2_), compared to fraction #1, had a stronger cytotoxic effect, which was most strongly observed against MO3.13 cells after 72 h of incubation. Due to the weaker effect of fractions #1 and #2 on glioblastoma cells compared to the physiological cell line, both fractions were excluded from further studies. Compared to fractions #1 and #2, the BV fraction containing melittin (#3) dramatically reduced the viability of all tested cells at a concentration of just a few μg/mL, for each of the incubation times tested. The IC_50_ values for fraction #3 against both glioblastoma cell lines were similar to the IC_50_ values of melittin standard, suggesting that cytotoxic properties of this fraction might came from melittin, which constituted approximately 70% of the dry weight of this fraction. Another interesting observation was the very low IC_50_ value for crude BV on MO3.13 cells incubated for 72 h. Crude BV showed a much stronger cytotoxic effect compared to fraction #3 and the melittin standard. The constituents present in fraction #2 (mostly PLA_2_), which were also present in crude BV, could be responsible for the observed effect, as a strong effect reducing the viability of the tested physiological cells was also observed for this fraction. This may indicate that other compounds apart from melittin may modify its action on certain cell types. Similar observations indicating higher cytotoxic potential of bee venom compared to melittin were described in relation to HCT116 colon cancer cell line [29].

Traditionally, the MMPs are associated with tumor progression, whereas a higher TIMPs level indicates better prognosis as these act as MMP inhibitors [30]. Thus, a decrease in enzyme expression with a simultaneous increase in the level of inhibitors seems to be a favorable scenario desired during treatment. However, studies also report an increased concentration of inhibitors in the case of very aggressive cancers. Research by Dibdiakova et al. showed high expression of MMP-2 and MMP-9 in human glioblastoma tissue compared to other types of intracranial tumors, simultaneously with very high expression of TIMP-1 (several times higher than in other types of tumors) [31]. Therefore, it is not clear whether the increase in the activity of specific MMPs is always unfavorable, as these enzymes can have opposing effects on different types of cancers [32]. This is observed, for example, in a study using MMP-9 gene knockout in an animal model; there was a lack of MMP-9-delayed tumorigenesis in the C3(1)-Tag model of basal-like triple-negative breast cancer, but there was no effect on tumorigenesis in the (MMTV)-Neu model of luminal breast cancer [33]. Therefore, the subtle role of MMPs and TIMPs is more complex than could be attributed solely to their ability to degrade connective tissue proteins.

Gelatin zymography is a sensitive, semi-quantitative method used for the detection and analysis of MMP-2 and MMP-9 in biological fluids and tissue homogenates [34]. MMP-2 and MMP-9 are enzymes associated with the extracellular matrix. By degrading extracellular proteins, both enzymes have the ability to model connective tissue and contribute, among others, to the development of the inflammatory process or tumor growth. The study revealed the presence of MMP-2 in the culture media of all tested cell lines. Moreover, in the medium collected from MO3.13 the presence of MMP-9 was also detected besides MMP-2. A dose-dependent reduction in the activity of both enzymes was observed for fraction #3, crude BV, and melittin for all incubation times used. Moreover, when analyzing zymographic gels, greater MMP-2 activity was observed (thicker and more distinct band) in the medium collected from the LN229 culture. Previously performed studies showed the presence of MMP-2 and MMP-9 in two other culture media of the glioblastoma line, GAMG and 8-MG-BA, and the activity of both enzymes was twice as high in the medium collected from 8-MG-BA [22]. The observation that the LN229 line has a greater ability to secrete MMP-2 compared to the LN18 line indicates that glioblastomas of different origins have various abilities to model the tissues surrounding the tumor. It is worth noting that the dose-dependent reduction in the activity of gelatinases should be explained by the reduced expression and secretion of these enzymes into cell culture media induced by all analyzed compounds. This result cannot be explained simply by the decreased cell viability, as cell death under the influence of fraction #3, crude BV, or melittin would lead to the release of intracellular reservoirs of enzymes into the medium. In such cases, it could even result in an increase in the activity of these enzymes.

TIMPs have the ability to inhibit the activity of MMPs by binding to the active center of enzymes and displacing water molecules necessary for catalysis [35]. During zymography, electrophoretic separation of the tested proteins occurs and the possible TIMPs present in the sample material are separated from the heavier MMP proteins (critical description of zymography [36]). Therefore, the ELISA method was used to evaluate TIMP levels in cell culture media. The study showed a dose- and incubation-time-dependent reduction in the concentration of TIMP-1 and TIMP-2 in the LN229 and MO3.13 cell lines with all analyzed compounds. At a concentration of 3.5 μg/mL, fraction #3, crude BV, and melittin reduced the level of both inhibitors almost completely. In the case of the LN18 glioblastoma line, fraction #3 at lower concentrations slightly increased the TIMP-1 level in the culture medium after 24 h, 48 h, and 72 h of incubation. At higher concentrations of fraction #3 (3.5 or 5.0 μg/mL, depending on incubation time), TIMP-1 levels were reduced. The effect of the fractions containing melittin on the TIMP-1 concentration was different compared to the effect of the melittin standard, which reduced the level of TIMP-1 in the LN18 cell medium at each of the analyzed incubation times, starting from the lowest concentrations. The different effects on the TIMP-1 level could be caused by the additional presence of other BV components within fraction #3 in addition to melittin. The level of TIMP-2 was reduced most rapidly by each of the tested compounds in the culture medium of the LN229 line at each of the incubation times tested.

The conducted studies have limitations. Firstly, far-reaching clinical conclusions should not be drawn based on studies conducted in vitro. The efficacy of BV in the treatment of cancers, including cancers located in the central nervous system, should be confirmed in animal studies and, above all, in a clinical trial. For example, in the case of in vitro studies, the problem of difficulties related to the delivery of BV to tumor cells typically located intracranially disappears. In order to obtain a high concentration of the drug at the target site, it would be necessary to consider either administering the drug in the form of an injection directly into the tumor or at least administering the drug directly into the cerebrospinal fluid, and counting on better penetration of the substance into the diseased tissue. Such methods of treatment are used in medicine. Biodegradable implants gradually releasing carmustine, a chemotherapeutic agent used in the treatment of glioblastoma, are placed directly in the postoperative bed, providing much better availability of the drug compared with other routes of administration [37]. Meanwhile, a synthetic peptide initially isolated from *Conus magus* is used in the treatment of neuropathic pain and is administered intrathecally [38]. Future studies on the treatment of glioma with BV may take into account the above solutions.

Parenteral administration of BV causes allergic reactions, which can even lead to anaphylactic shock. Melittin also has high toxicity and a strong hemolytic effect, which negatively affects the potential use of BV at high doses [39]. In this case, strategies are proposed to modify the structure of melittin or create conjugates of melittin with other molecules [40]. Chemical modifications in order to reduce the toxicity of BV include conjugation with polyethylene glycol (PEGylation), truncation, and peptide synthesis with dextrogenous (d-)amino acids. These strategies could potentially allow higher concentrations of apitoxin to be used for treatment [41]. In order to reduce the direct interaction of melittin with erythrocyte membranes, encapsulation of this compound in nanoparticles has also been considered [42]. A promising strategy for attenuating the hemolytic activity of melittin is also its conjugation with aptamers [43].

One way to reduce the negative effects of natural peptides while increasing their therapeutic potential is to modify their structure. Previous research on snake venom PLA_2_ indicates that its synthesized 13 amino C-terminal fragments with substituted phenylalanine instead of leucine may damage bacterial cell membranes while maintaining the integrity of the erythrocyte membrane [44]. Another study, also concerning peptides obtained on the basis of PLA_2_ snake venom, showed the effect of these compounds not only on bacterial cell membranes, but also on eukaryotic cells of selected tumor lines (all peptides showed a cytotoxic effect on breast adenocarcinoma MCF-7 line, and one of them on colon adenocarcinoma Caco-2 line). At the same time, all tested peptides showed a negligible tendency (below 3%) to induce hemolysis [45]. The mechanism of action of C-terminal PLA_2_ peptides was discussed in detail in the publication by Lomonte et al. [46]. Although the discussed agents also demonstrate membrane activity towards physiological cells, the authors of the publication indicate that, for example, the modified C-terminal peptide pEM-2 (D-enantiomer) showed an antitumor effect in vivo towards ETM2 breast cancer cells comparable to the effect exerted by paclitaxel [46]. Similar studies on membrane interactions have also been performed on modified melittin derivatives [47]. However, it must be remembered that various types of modification of natural compounds may reduce their cytotoxic properties towards glioblastoma cells.

Secondly, a certain limitation of the study is the evaluation of MMPs and TIMPs only at the protein level. Although these compounds demonstrate biological activity as protein molecules, the evaluation of expression at the mRNA level would show whether BV components stimulate the synthesis of new protein molecules or only lead to the secretion of these proteins outside the cells (to explore the problem of mRNA/protein correlation see [48]). Thirdly, the study concerns the effect of BV or its components, and does not take into account the possible synergistic effect of these components with the basic anti-glioma drug temozolomide [49].

Cancer treatment with BV requires further research. As with other substances of natural origin, it is difficult to determine which of the components exerts the therapeutic effect and what the mechanism of its action is. In addition, there may be a network of connections between the components of the venom that affect their biological activity. Such interaction of components is emphasized by some researchers in the case of extracts derived from hemp. This is the so-called entourage effect, which still remains a hypothesis [50]. The method of obtaining BV may also have an impact on its biological effect. Venom obtained by the commonly used electroconvulsive method may differ in composition from venom injected during stinging. Volatile components of BV may evaporate during collection by electroconvulsive means, and venom injected by a bee may contain proteins derived from glandular tissue, changing its therapeutic nature [51].

## 4. Materials and Methods

### 4.1. Reference Standards and Chemicals

Apamin ≥95%, phospholipase A_2_ 600–2400 units/mg, melittin ≥85% standards, trifluoroacetic acid (TFA) (≥99%), HPLC-grade methanol, and acetonitrile were purchased from Sigma-Aldrich (St. Louis, MO, USA). Water for HPLC was purified by Ultrapure Millipore Direct-Q^®^3UV–R (Merck Millipore, Billerica, MA, USA).

### 4.2. Bee Venom Fractionation

A total of 1 g of commercially available, dried BV powder obtained from *Apis mellifera* (https://www.beevenomlab.com) was dissolved in 50 mL of deionized water, centrifuged, and the resulting supernatant was filtered through a membrane filter. The solution was fractionated on a column packed with 35 g of LiChroprep™ RP-18 sorbent (40–63 µm) (Merck, Darmstadt, Germany). The venom components were eluted with aqueous methanol solutions (from 5% to 100% MeOH) and 16 sub-fractions were obtained. The obtained eluates were analyzed by HPLC and fractions with similar quality profiles were combined to obtain 3 fractions. The methanol–water fractions were concentrated on a rotary evaporator (Heidolph Hei-Vap Expert, Heidolph Instruments GmbH & Co. KG, Schwabach, Germany) (150 mbar; 50 °C), frozen at −80 °C, and then dried by lyophilization (freeze dryer Christ Alpha 2–4 LDplus, Christ, Osterode am Harz, Germany; 0.001 mbar for 2 days).

### 4.3. HPLC-PDA Analysis

The chromatographic analyses were performed on a VWR Hitachi Elite LaChrom HPLC system equipped with a photodiode-array detector (UV–vis, PDA) (190–350 nm) and EZChrom Elite software version 3.3.2 (Merck, Darmstadt, Germany). The chromatographic system for the analysis was as follows: an RP18 reversed-phase column Kinetex (Phenomenex, Torrance, CA, USA) (10 cm × 4.6 mm i.d., 2.6 μm particle size), 20 °C, and a flow rate of 1 mL/min. A mixture of acetonitrile with 0.025% of TFA (solvent A) and water with 0.025% of TFA (solvent B) were used as a mobile phase. The compounds were separated using gradient elution with the following program: 0–5 min 10% A, 90% B; 5–25 min 10–50% A, 90–50% B; 25–40 min 50% A, 50% B; and 40–65 min 50–100% A, 50–0% B. Data were collected between 190 and 300 nm. The identity of the compounds was established via a comparison of the retention times and PDA spectra with the corresponding standards. Quantitative analysis was performed at λ = 195 nm for apamin, phospholipase A_2_, and melittin. The composition of the sub-fractions was checked directly in the eluate (injection volume 10 µL). The content of major BV components in the final fractions was determined by dissolving an accurately weighed fraction (approximately 1 mg) in 1 mL of methanol (injection volume 5 µL).

### 4.4. Cell Culture

Two human glioblastoma cell lines (LN229—ATCC CRL-2611; LN18—ATCC CRL-2610) were used in the study. MO3.13 cells (TebuBio, Lier, Belgium), an immortal human–human hybrid cell line that express phenotypic characteristics of primary oligodendrocytes, were used as a control. The cells were cultured in Dulbecco’s modified Eagle medium (DMEM) supplemented with 10% (*v*/*v*) fetal bovine serum, penicillin (10,000 U/mL), and streptomycin (10 mg/mL). The cells were incubated at 37 °C, 5% CO_2_ atmosphere. The cells were maintained in the logarithmic growth phase by regular passage at 80% confluence.

### 4.5. Cell Viabillity Assay

After 24 h of incubation in growth medium with an addition of 10% fetal bovine serum (FBS) on 96-well plates, the cells were treated with an increasing concentration of BV and BV fractions. The experiment started with the concentrations of 0.5, 1.0, 1.5, 2.0, 2.5, 3.5, 5.0, and 7.5 μg/mL for BV and the three BV fractions obtained. The concentrations were selected on the basis of other studies available in the literature [52]. Because fraction #1 (containing apamin) and fraction #2 (containing phospholipase A_2_) did not show significant cytotoxic activities at these doses, higher concentrations were used to assess their effect on the tested cells: 10, 25, 35, 50, 65, 75, 85, and 100 μg/mL. The activity of fraction #3 (containing melittin) was compared with commercially available melittin standard (Sigma-Aldrich, Cat No. 4446605).

The cells were cultured at 37 °C in the presence of 5% CO_2_–air for the next 24 h, 48 h, and 72 h. The cytotoxicity of BV and BV fractions was evaluated using the MTT colorimetric method which is based on the ability of viable cells to transform yellow, soluble tetrazolium salts [3-(4,5-dimethylthiazol-2-yl)-2,5-diphenyltetrazolium bromide, MTT] into purple, insoluble formazan, by cellular dehydrogenases. After incubation, the cell cultures were supplemented with 10 μL per well of 5 mg/mL MTT (Sigma-Aldrich, Saint Louis, MO, USA) stock in PBS, and the incubation was continued for 4 h at 37 °C. Next, the medium with MTT was removed, and the formed crystals were dissolved in 100 μL of DMSO. The solution absorbance was measured at 570 nm, using a spectrophotometric plate reader from Epoch, BioTek Instruments (Winooski, VT, USA). The relative cytotoxic activity was determined as the amount of BV or BV fractions capable of reducing 50% of cell viability (IC_50_ value). The experiment was performed four times with six replicates for each concentration.

### 4.6. Zymography

After 24 h, 48 h, and 72 h of incubation with BV concentrations of 1.0, 2.0, 3.5, and 5.0 μg/mL vs. vehicle, the media from the cells were collected to measure the secreted MMP-2 and MMP-9 activities. The analysis was performed for fraction #3 and compared to crude BV and melittin standard.

The experiment was performed three times (*n* = 3) for each concentration of analyzed compounds. MMP-2 and MMP-9 activities were evaluated with the use of gelatin zymography according to previously applied methods [53]. Briefly, 80 μL of cell culture media was mixed with 20 μL of sample loading buffer containing 10% sodium dodecyl sulfate (SDS) and incubated for 30 min at room temp. Next, the proteins were separated by polyacrylamide gel electrophoresis (PAGE) on a 10% gel supplemented with 0.05% gelatin type A from porcine skin; G2500 (Sigma-Aldrich, St. Louis, MO, USA). After electrophoresis, the gels were washed with 2.5% Triton X-100 three times for 20 min each to remove SDS. Next, 20 h of incubation was performed at 37 °C in a buffer of pH 7.2 containing 1% Triton X-100. The gels were stained with a solution containing 0.1% Coomassie Blue R-250, 20% methanol, and 10% glacial acetic acid in distilled water and destained in a 10% solution of acetic acid thereafter. The MMP-2 and MMP-9 were detected as colorless bands (digested gelatin) on a blue background (undigested gelatin). Zymography allows detecting both pro-form (latent) and active forms of MMPs as the SDS is used to activate non-proteolytic pro-MMPs into MMPs with catalytical activity without changing their molecular mass. The enzymes were identified by comparing their localization with the localization of the enzyme standards of MMP-2 and MMP-9 (R&D Systems).

Zymographic gels were scanned and quantified with ImageJ software, version 1.41 (National Institute of Health, Bethesda, MD, USA). The activities of MMP-2 and MMP-9 were expressed as the optical density (OD) of the substrate lysis zone.

### 4.7. ELISA

Commercially available ELISA kits (R&D Systems, Minneapolis, MI, USA) were applied to determine TIMP-1 and TIMP-2 in the cell medium. All procedures were performed in accordance with the manufacturer manual. The experiments were conducted three times and the mean values of the three repetitions were taken as final results.

### 4.8. Statistics

The results of cell viability were expressed as mean and standard deviation (SD). The statistical significance of the differences between the control vehicle vs. groups incubated with increasing concentration of analyzed compounds through 24, 48, and 72 h was evaluated using GraphPad Prism v10 software (Boston, MA, USA). Non-linear regression (curve fit) was used to establish the IC_50_ values of the analyzed compounds after 24, 48, and 72 h of incubation for all cell lines assessed, using “Quest Graph™ IC50 Calculator” (AAT Bioquest, Inc., Pleasanton, CA, USA, https://www.aatbio.com/tools/ic50-calculator, accessed on 15 January 2024). Values were considered significant if *p* < 0.05.

## 5. Conclusions

Bee venom has cytotoxic effects not only on selected glioblastoma cell lines, but also on physiological cells. The cytotoxicity of BV is mainly due to the melittin present in it, but other compounds present in BV may modify the effect of melittin on certain cell types. The anti-tumor effect of BV components may result from reducing the secretion of MMP-2 and MMP-9 into the extracellular space, thus reducing the potential ability to degrade the tissue surrounding the tumor. Further research on the effect of bee venom should include analysis of its effect on other types of glioblastoma cells as well as initiation of research on in vivo models.

## Figures and Tables

**Table 1:** Percentage content of the main components of bee venom in the obtained fractions. The presence of apamin was confirmed only in fraction #1, phospholipase A_2_ in fraction #2, and melittin in fraction #3.

Compound	Fraction #1	Fraction #2	Fraction #3
Apamin	8.44 ± 1.00	n.d.	n.d.
Phospholipase A_2_	n.d.	95.76 ± 1.53	n.d.
Melittin	n.d.	n.d.	69.34 ± 0.58

n.d., not detected.

## Data Availability

The data presented in this study are available on request from the corresponding author.

## Supplementary Materials

The following supporting information can be downloaded at: https://www.mdpi.com/article/10.3390/ijms26062376/s1.
