# Supplementary material for: Anticancer Activity of Melittin-Containing Bee Venom Fraction Against Glioblastoma Cells In Vitro
Source: Int J Mol Sci. 2025 Mar 7;26(6):2376. doi: 10.3390/ijms26062376 (PMC11942036; doi:10.3390/ijms26062376)
Supplement: Supplementary file 1 [file ijms-26-02376-s001.zip › ijms-3483086-supplementary.pdf]

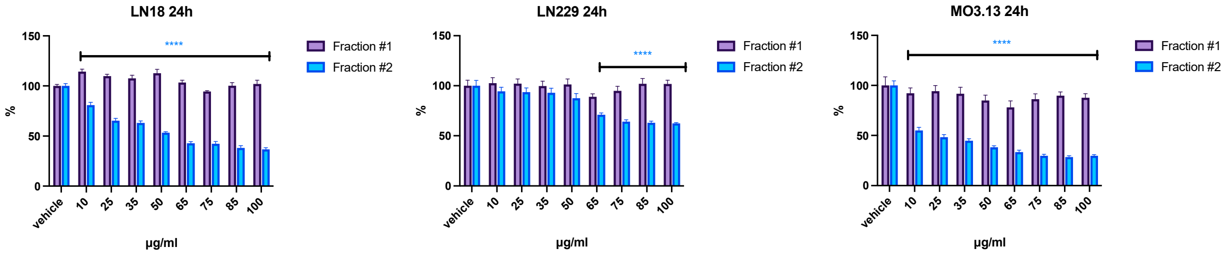

Figure S1. The effect of fraction #1 and fraction #2 on LN18, LN229, MO3.13 cell lines viability after **24 h** of incubation. Vehicle were set as 100% ± SEM. ANOVA multiple comparison *post-hoc* test. **** $p < 0.0001$ compared to controls (vehicle).

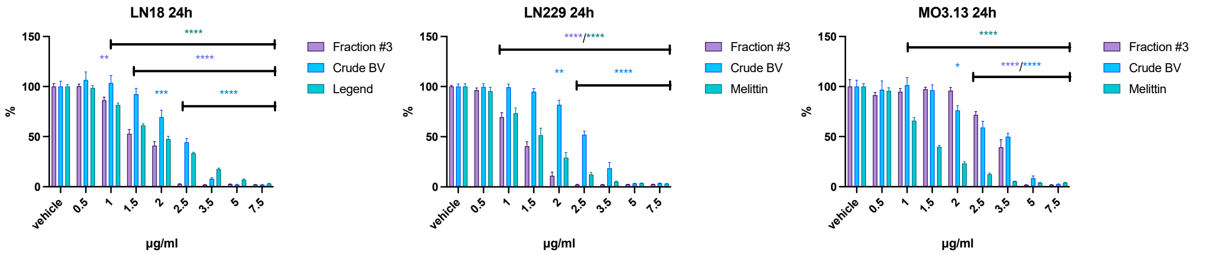

Figure S2. The effect of fraction #3, crude BV and melittin standard on LN18, LN229, MO3.13 cell lines viability after **24 h** of incubation. Vehicle were set as 100% ± SEM. ANOVA multiple comparison *post-hoc* test. * $p < 0.05$, ** $p < 0.01$, *** $p < 0.001$, ****$p<0.0001$ compared to controls (vehicle).

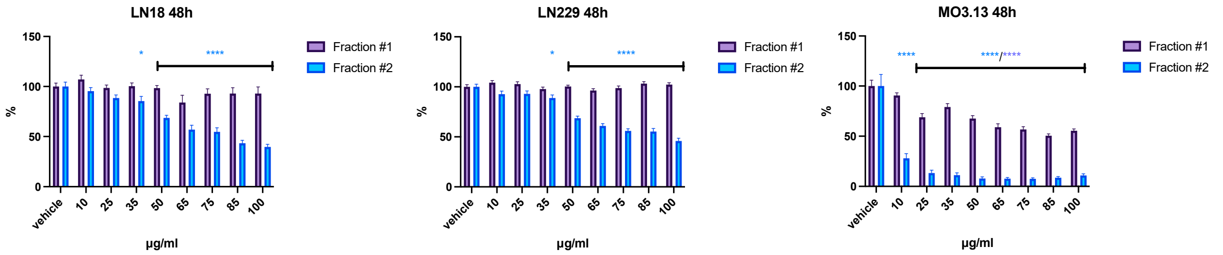

Figure S3. The effect of fraction #1 and fraction #2 on LN18, LN229, MO3.13 cell lines viability after **48 h** of incubation. Vehicle were set as 100% ± SEM. ANOVA multiple comparison *post-hoc* test. * $p < 0.05$, **** $p < 0.0001$ compared to controls (vehicle).

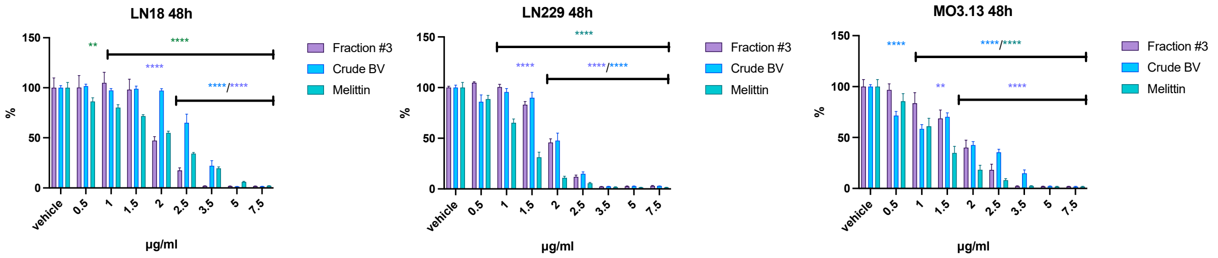

Figure S4. The effect of fraction #3, crude BV and melittin standard on LN18, LN229, MO3.13 cell lines viability after **48 h** of incubation. ANOVA multiple comparison *post-hoc* test. ** $p < 0.01$, **** $p < 0.0001$ compared to controls (vehicle).

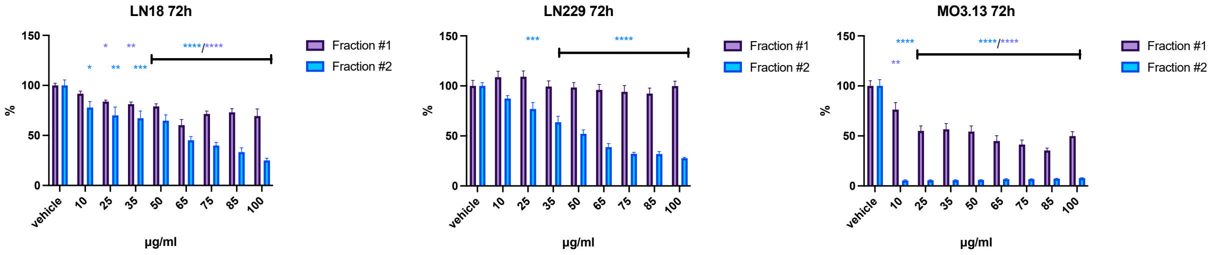

Figure S5. The effect of fraction #1 and fraction #2 on LN18, LN229, MO3.13 cell lines viability after **72 h** of incubation. Vehicle were set as 100% ± SEM. ANOVA multiple comparison *post-hoc* test. * $p < 0.05$, ** $p < 0.01$, *** $p < 0.001$, **** $p < 0.0001$ compared to controls (vehicle).

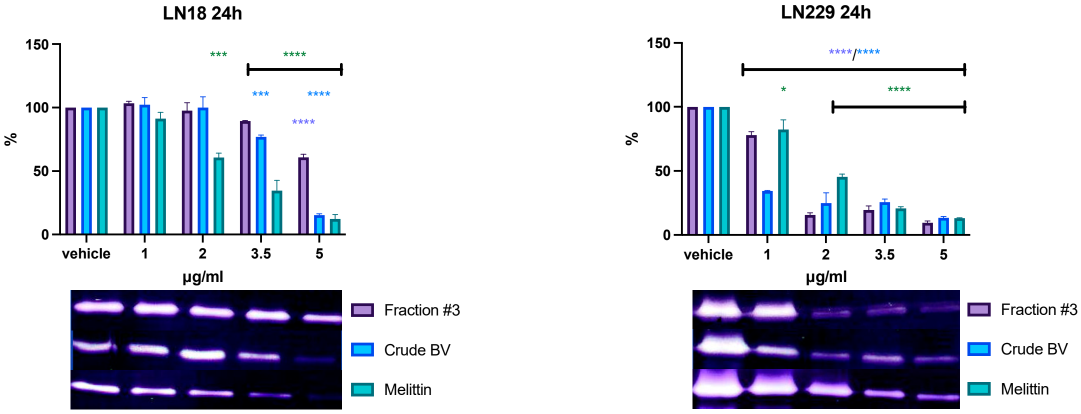

Figure S6. The effect of fraction #3, crude BV and melittin standard on **MMP-2** secretion from LN18 and LN229 cell lines after **24 h** of incubation. Enzyme activities were expressed as % of measured optical density and it was set as 100% for vehicle ± SEM. Figures are shown together with representative zymograms. ANOVA multiple comparison *post-hoc* test. * $p < 0.05$, *** $p < 0.001$, **** $p < 0.0001$ compared to controls (vehicle).

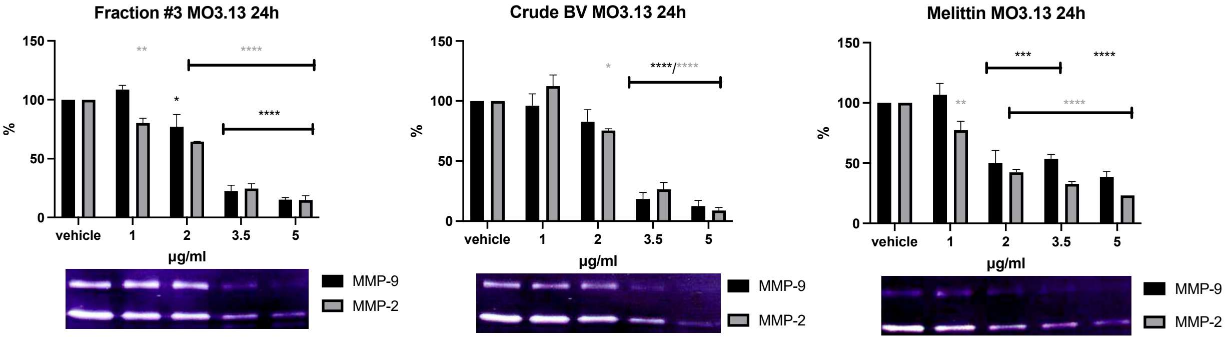

Figure S7. The effect of fraction #3, crude BV and melittin standard on **MMP-9** and **MMP-2** secretion from MO3.13 cell line after **24 h** of incubation. Enzyme activities were expressed as % of measured optical density and it was set as 100% for vehicle ± SEM. Figures are shown together with representative zymograms. ANOVA multiple comparison *post-hoc* test. * $p < 0.05$, ** $p < 0.01$, *** $p < 0.001$, **** $p < 0.0001$ compared to controls (vehicle).

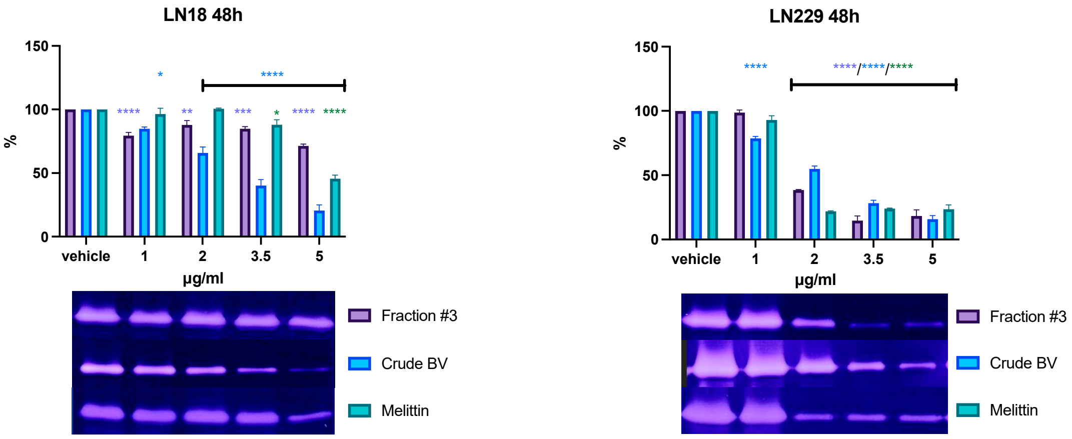

Figure S8. The effect of fraction #3, crude BV and melittin standard on **MMP-2** secretion from LN18 and LN229 cell lines after **48 h** of incubation. Enzyme activities were expressed as % of measured optical density and it was set as 100% for vehicle ± SEM. Figures are shown together with representative zymograms. ANOVA multiple comparison *post-hoc* test. * $p < 0.05$, ** $p < 0.01$, *** $p < 0.001$, **** $p < 0.0001$ compared to controls (vehicle).

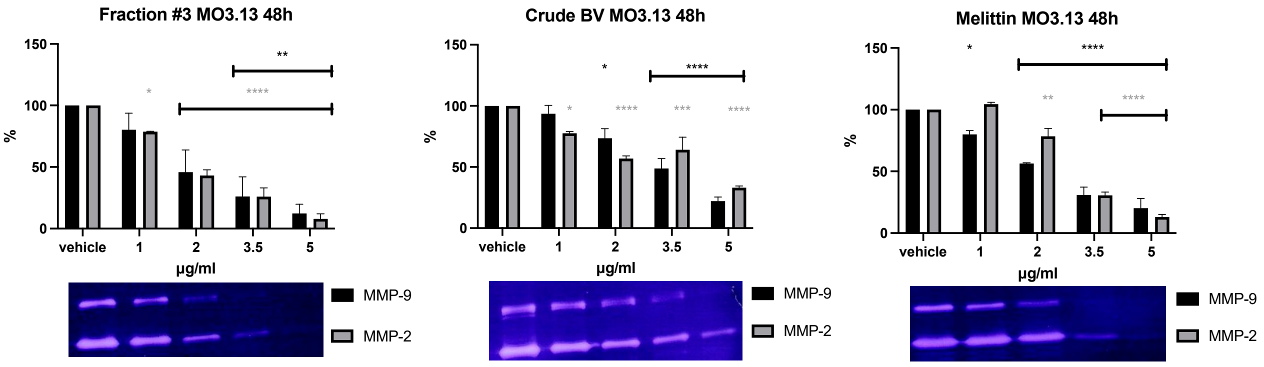

Figure S9. The effect of fraction #3, crude BV and melittin standard on **MMP-9** and **MMP-2** secretion from MO3.13 cell line after **48 h** of incubation. Enzyme activities were expressed as % of measured optical density and it was set as 100% for vehicle ± SEM. Figures are shown together with representative zymograms. ANOVA multiple comparison *post-hoc* test. * $p < 0.05$, ** $p < 0.01$, *** $p < 0.001$, **** $p < 0.0001$ compared to controls (vehicle).

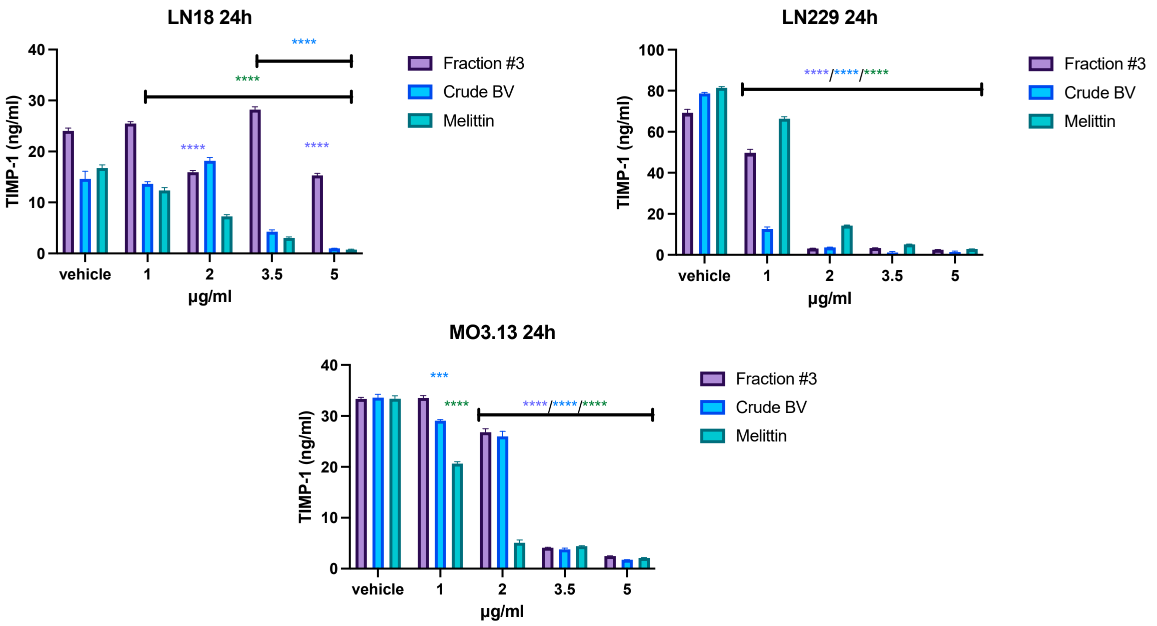

Figure S10. The effect of fraction #3, crude BV and melittin on **TIMP-1** secretion from LN18, LN229 and MO3.13 cell lines after **24 h** of incubation. Mean TIMP-1 level ± SEM. ANOVA with multiple comparison *post-hoc* test. *** $p < 0.001$, **** $p < 0.0001$ compared to controls (vehicle).

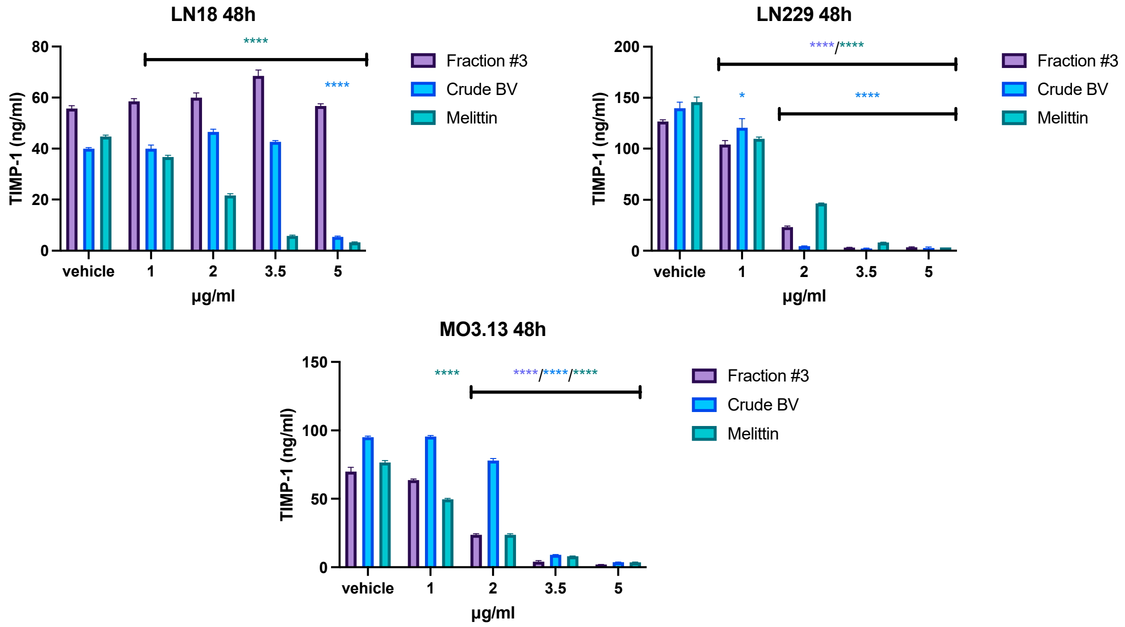

Figure S11. The effect of fraction #3, crude BV and melittin on **TIMP-1** secretion from LN18, LN229 and MO3.13 cell lines after **48 h** of incubation. Mean TIMP-1 level ± SEM. ANOVA with multiple comparison *post-hoc* test. * $p < 0.05$, **** $p < 0.0001$ compared to controls (vehicle).

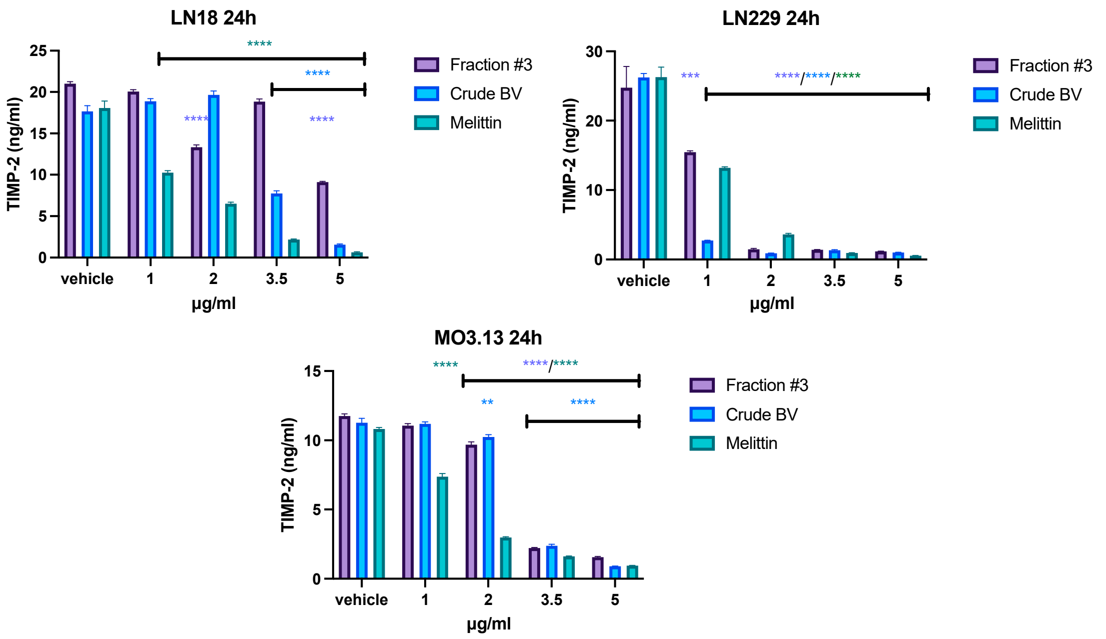

Figure S12. The effect of fraction #3, crude BV and melittin on **TIMP-2** secretion from LN18, LN229 and MO3.13 cell lines after **24 h** of incubation. Mean TIMP-2 level ± SEM. ANOVA with multiple comparison *post-hoc* test. ** $p < 0.01$, *** $p < 0.001$, **** $p < 0.0001$ compared to controls (vehicle).

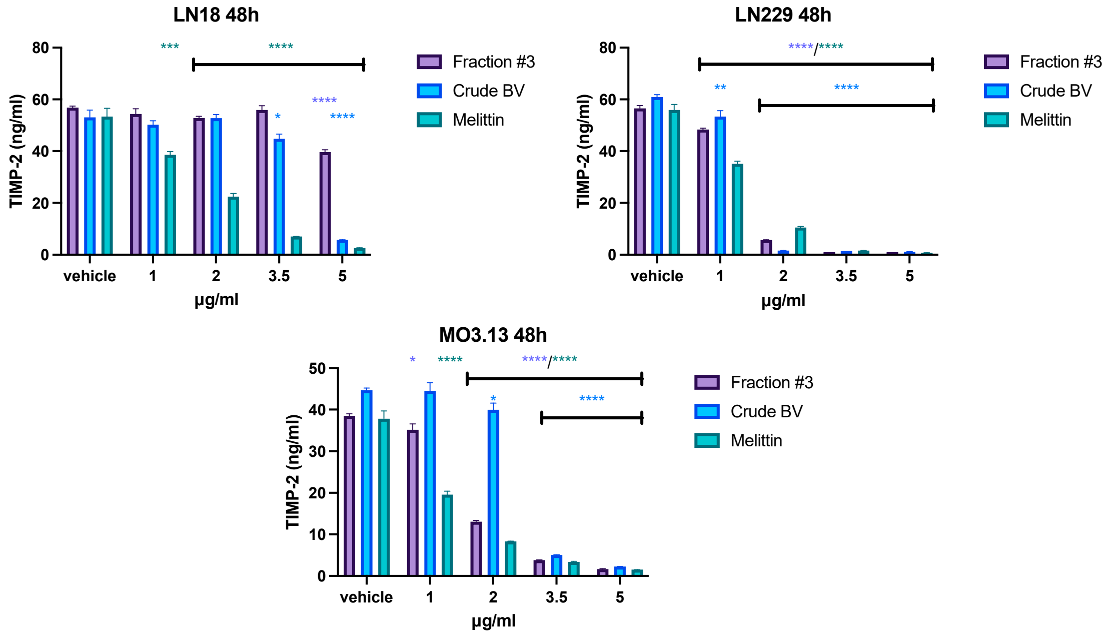

Figure S13. The effect of fraction #3, crude BV and melittin on **TIMP-2** secretion from LN18, LN229 and MO3.13 cell lines after **48 h** of incubation. Mean TIMP-2 level ± SEM. ANOVA with multiple comparison *post-hoc* test. * $p < 0.05$, ** $p < 0.01$, *** $p < 0.001$, **** $p < 0.0001$ compared to controls (vehicle).

Table S1. IC_{50} values of fraction #3, crude BV and melittin standard obtained for evaluated cell lines after 24, 48 and 72 h of incubation. Values were expressed in µg/ml.

Cell line	Fraction #3	Crude BV	Melittin
Incubation 24 h			
LN18	1.58	2.26	1.81
LN229	1.26	2.54	1.42
MO3.13	3.14	2.95	1.25
Incubation 48 h			
LN18	1.97	2.79	1.97
LN229	1.87	1.96	1.16
MO3.13	1.74	1.49	1.13
Incubation 72 h			
LN18	2.22	3.32	2.07
LN229	1.40	2.55	0.87
MO3.13	1.00	0.10	2.32
